# The First Reported Foodborne Botulism Outbreak in Riyadh, Saudi Arabia: Lessons Learned

**DOI:** 10.1007/s44197-024-00255-z

**Published:** 2024-06-05

**Authors:** Nadeem Gul Dar, Sarah H. Alfaraj, Khulood Naser Alboqmy, Nazia Khanum, Faleh Alshakrah, Hassan Abdallah, Mohammad Hosni Badawi, Ohoud Mohammed Alharbi, Khadijh Ahmed Alshiekh, Abdullah M Alsallum, Ahmed Hassan Shrahili, Zeidan A Zeidan, Zaki Abdallah, Ahmed Ali Majrashi, Ziad A. Memish

**Affiliations:** 1 Prevention and Control of Infection Administration, King Saud Medical city, Riyadh, Saudi Arabia; 2 King Salman Hospital, Riyadh, Saudi Arabia; 3 Imam Abdulrahman Alfaisal Hospital, Riyadh, Saudi Arabia; 4 Al Iman General Hospital, Riyadh, Saudi Arabia; 5 Research and Innovation Center, King Saud Medical City, Riyadh, Saudi Arabia; 6 College of Medicine, Al Faisal University, Riyadh, Saudi Arabia; 7 Hubert Department of Global Health, Rollins School of Public Health, Emory, University, Atlanta, USA

**Keywords:** Food Poisoning, Botulism, Saudi Arabia, Outbreak Investigation

## Abstract

**Background:**

Botulism has not been previously reported in the Kingdom of Saudi Arabia. This rare and sometimes fatal foodborne illness is caused by neurotoxins and primarily results from consuming home-canned fruits, vegetables, dairy, and seafood products & it can lead to paralysis.

**Objective:**

The purpose of this study was to evaluate the clinical features of patients who developed botulism in Riyadh in 2024 after consuming mayonnaise from a well-known local chain of restaurants in Riyadh, Saudi Arabia.

**Methods:**

We conducted a retrospective analysis of medical records and interviewed patients or their attendants for all hospitalized cases of foodborne botulism at Riyadh First Health Cluster. For each patient, a standard case report form was completed, containing information on demographics, clinical aspects, botulinum test results, and type of exposure. Descriptive statistics were applied to assess the data. During the outbreak, nineteen patients with foodborne diseases were admitted to Riyadh First Health Cluster Hospitals. Following thorough physical examinations, botulism was suspected in each case.

**Results:**

Eight of the 19 suspected foodborne illness patients fully satisfied the botulism case definition requirements set forth by the Saudi Arabian Public Health Authority (Weqaya). Among these eight patients, 2 (25%) were male and 6 (75%) were female, with a mean age of 23.25 ± 9.29 years (range: 12–38 years). The incubation period for our patients was 36.25 ± 26.26 h. Notable symptoms included dysphagia in all eight patients (100%), dysarthria, generalized weakness, nausea and vomiting in seven patients (88%), diplopia in four patients (50%), and stomach discomfort in three patients (38%). Of the eight cases, six required intubation, one mimicked brain death, and two were stable. The presence of Clostridium botulinum spores as the cause of the outbreak was confirmed by detecting botulinum spores in contaminated food.

**Conclusion:**

Diplopia and dysarthria were the most common early sign of botulism. Early manifestations may include respiratory symptoms without any musculoskeletal symptoms. or nausea, vomiting and disorientation.

## Introduction

In the Kingdom of Saudi Arabia, foodborne botulism has never been documented. Foodborne botulism is an uncommon yet deadly disease, affecting both humans and other vertebrates. The underlying cause is botulinum neurotoxin, which is considered one of the most lethal substances humans have ever encountered [1, 2]. The predominant bacterium that produces this toxin is Clostridium botulinum, rarely strains of *Clostridium baratii* and *Clostridium butyricum*, a gram-positive bacillus, anaerobic, spore-forming that developed in harsh environments to promote survival. Normally, spores pass through healthy human digestive system without causing any disease, except for infants due to immature intestine and underdeveloped gut flora [3, 4].

Traditionally, botulinum neurotoxins have been classified into at least seven serotypes, denoted by the letters A through G. Notably, a novel eighth serotype was identified in 2016 [5, 6]. Among these, serotypes A, B, E, and more rarely F cause human disease. Serotype A is associated with the most severe clinical manifestations, with a higher proportion of patients requiring mechanical ventilation for respiratory support. On the other hand, serotype B typically manifests as a milder disease compared to serotype A [7].

Botulinum toxin affects human body with neuroparalytic illness characterized by flaccid paralysis of the motor and autonomic nerves that descend, starting with the cranial nerves. These symptoms can include dysphagia, muscle weakness, diplopia, ptosis, blurred vision, slurred speech, respiratory distress or failure, and ocular palsy. Despite the characteristic features of botulism, including symptoms that typically begin in the cranial nerves, consistent descending progression, symmetry, and absence of sensory nerve dysfunction, it is frequently misdiagnosed as Guillain-Barre syndrome, Miller-Fisher syndrome, myasthenia gravis, or other central nervous system diseases [1, 2, 7].

Human botulism comes in various forms, including foodborne, wound, infant, unclassified (adult intestinal colonization), iatrogenic (medical treatment complications), and inhalation botulism. Despite having different origins, all types of botulism present with a common clinical presentation that is typified by neuromuscular paralysis caused by the toxin. Particularly, ingesting preformed botulinum neurotoxin complexes found in contaminated food is the cause of foodborne botulism, which is the most common type [8–11].

Foodborne botulism exhibits a variable incubation period affected by toxin type and quantity digested, ranging from a minimum of 4 h to a maximum of 8 days after ingesting toxin-contaminated food. In most cases presentation occurs within a window of 12 to 36 h. Patients diagnosed with botulism are not considered contagious. Therefore, beyond standard precautions, no additional isolation measures are necessary [8, 9, 12, 13]. The surveillance system for foodborne illness in Riyadh involves the systematic collection, analysis, and reporting of data on foodborne disease cases to detect outbreaks, identify sources of contamination, and implement public health interventions to prevent further spread. This system ensures timely monitoring and response to potential food safety threats, enhancing overall public health protection. To our knowledge, this is the first report to describe a clinicopathological outbreak of botulism involving multiple centers within Riyadh First Health Cluster (5 Hospitals with bed capacity of 2302). The aim of this study is to report on the clinical and epidemiological characteristics of the 19 individuals who were suspected and the 8 cases that met the case definition for botulism who presented to our health cluster in the recent food-borne outbreak affecting 75 cases in total in Riyadh, KSA.

## Methodology

All patients referred to Riyadh First Health Cluster Hospitals between April 22–25, 2024, with gastrointestinal symptoms, respiratory distress, or descending paralysis after consuming mayonnaise from a popular Riyadh burger restaurant chain were admitted for assessment and care. The inclusion criteria, as provided by the Public Health Authority of Saudi Arabia, encompassed patients of both sexes and all ages who showed signs of respiratory distress, descending paralysis, or gastrointestinal distress within 72 h of consuming contaminated food. Exclusion criteria included individuals with pre-existing neurological disorders, a history of food allergies, or those who did not consume the contaminated food.

We conducted a retrospective analysis of medical records and interviewed patients, or their attendants hospitalized with foodborne botulism at Riyadh First Health Cluster. A standard case report form was completed for each patient, containing information on demographics, clinical aspects, botulinum test results, and type of exposure. Descriptive statistics were applied to assess the data.

Statistical analysis: Descriptive statistics, encompassing frequency distributions, were generated for all study variables using a recent statistical software package (Google sheets, Microsoft Excel).

Characteristics of a probable botulism case: In this study, a patient was deemed a likely case of botulism if they had a history of eating at a burger restaurant and showed symptoms of bilateral cranial nerve neuropathy, such as diplopia, impaired vision, dysphagia, bulbar paresthesia, and/or symmetrical weakness in peripheral muscles, as reported to the Saudi Public Health Authority.

Sample Gathering: Within 12 to 120 h of consuming contaminated food, biologic samples, such as feces, gastrointestinal contents, whole blood, and serum, were taken from each patient for laboratory investigation. The samples included:

A. 50 g of stool samples in a suitable container.
B. 25 mL of gastric contents, either vomitus or aspiration.
C. High rectal washout in cases where collecting stool samples was challenging.
D. 15 mL serum sample (serum collection should occur prior to administering antitoxin).

Furthermore, food samples were gathered by the Saudi Arabian government’s official authority to verify food contamination.

Data gathering: A group of nurses, epidemiologists, and healthcare specialists with expertise in infection control collected the data. The procedure included clinical interviews and patient physical examinations. A designated healthcare provider was responsible for gathering thorough clinical data, interviewing, and examining each patient or patient attendant individually. Conducting interviews and examinations at the time of admission and during the patient’s hospital stay facilitated ongoing evaluations of symptoms and clinical progress. Additionally, history was collected from the patients’ relatives or friends who had eaten the same food but did not develop symptoms. These asymptomatic individuals were followed up by our public health staff to monitor for the development of any botulism-related symptoms.

## Results

**Table 1:** Demographic details and characteristics of the foodborne botulism cases (*N* = 8), Saudi Arabia, 2024

characteristics		Total (%)	(%)
Age Group	≤ 18 years	3	38%
	18–29 years	3	38%
	30–39 years	2	25%
Sex	Male	2	25%
	Female	6	75%
Nationality	Saudi	7	87.5%
	Non - Saudi	1	12.5%
Incubation Period		20 to 105 h	
Mean age		23.25	ST 9.29

**Table 2:** Distribution of studied patients’ group according to clinical assessment on admission (*N* = 8), Saudi Arabia 2024

Clinical Manifestations	Dysphagia	8	100%
	Dysarthria	7	88%
	Generalized weakness	7	88%
	Nausea, vomiting	7	88%
	Double vision	4	50%
	Abdominal pain	3	38%
	Ptosis	1	13%
	Diarrhea	1	13%
Respiratory support required	Intubated	6	75%
	Room Air	2	25%
Motor Power	Upper extremities	3	38%
	Lower extremities	2	25%

### Demographic Data

A total of eight confirmed botulism cases were reported between April 22–25, 2024, with the peak occurring on April 22 (seven cases). The mean age of admitted patients was 23.25 ± 9.29 years (range: 12–38 years). Of these patients, 2 (25%) were male, and 6 (75%) were female. All cases presented to the hospitals between 20 and 105 h after eating contaminated food, with a mean presentation time of 36.25 ± 26.26 h (Table 1). All eight cases had the same epidemiological link, having eaten from different branches of a well-known local burger restaurant chain in Riyadh. In addition to these eight cases, three accompanying individuals (family/friends) who ate the same food did not develop botulism-specific symptoms. One of the eight cases was a patient’s daughter who had the same food, experienced similar symptoms, required intubation, and had a negative test result for botulism.

The most commonly reported signs and symptoms at hospital admission included dysphagia in all eight patients (100%), dysarthria, generalized weakness, nausea and vomiting in seven patients (88%), diplopia in four patients (50%), and stomach discomfort in three patients (38%). (Table 2).

Outcome: Of the eight cases meeting the case definition, only one was laboratory-confirmed. Two cases were stable, six required intubations, with one of them being brain dead. All the cases eventually recovered, and no more deaths were reported in our study.

## Discussion

This outbreak was traced to a small number of individuals who had consumed mayonnaise from a well-known group of a local chain of burger restaurant. Since serum samples were taken from patients at the time of admission and many patients were admitted to the hospital a few days after the onset of symptoms, the lengthy interval between the consumption of tainted food and the withdrawal of serum samples may account for these unfavorable outcomes. A number of variables, including the time and method of sample collection, the amount of toxin consumed, the rate at which the toxin enters the bloodstream, and the extravascular compartment’s uptake of the toxin, might lead to false-negative results in patients [14]. Additionally, it has been noted that a lot of clinical specimens had low toxin levels, which cannot show up for four days [15]. Furthermore, prior to the collection of samples for testing, all patients were administered anti-toxin, which explains the false negative result. The CDC recommends that serum specimens be obtained prior to administering botulism antitoxin because the antitoxin neutralizes the botulinum toxin, which can result in tests falsely indicating the absence of the toxin [16]. Thus, it can be said that clinical signs in patients who may have botulism are the most reliable indicator for a doctor to diagnose the disease rather than using laboratory markers.

Past medical and surgical history was taken to exclude neurological diseases, previous stroke, and other gastrointestinal problems. All patient’s symptomsstarted to appear after eating the meals from a specific restaurant. All the cases presented to the hospital from 20 h up to 5 days after ingestion of the contaminated food with a mean value of 36.25 ± 26.26 h. Systematic review, reported a median duration between exposure and symptom onset as approximately 1 day [17, 18]. This difference is expected due to the long incubation period of foodborne botulism which would reach up to 8 days.

Botulism has specific clinical symptoms, but diagnosing it requires a high index of suspicion. Because the symptoms mostly affect the gastrointestinal tract and central nervous systems, this case series is similar to those that have been published elsewhere [19–22]. These specific symptoms with epidemiological link can be used for early recognition of foodborne botulism cases with further early botulinum antitoxin administration and other supportive care reduces the degree and severity of paralysis, sometimes preventing the development of respiratory compromise and reducing the length of time patients require mechanical ventilation and intensive care.

The fact that nearly 50% of suspected cases confirmed with botulism reflects the severity of clinical prodromes in persons with botulism. Out of the eight cases, two were stable, six required intubations, and one among 6 intubated is mimicking brain death. The attack rate is not 100%, as was shown in previous foodborne botulism outbreaks (23–24). In our investigation, only eight of the eleven participants who consumed the identical meal from the same local burger restaurant displayed signs of botulinum toxicity. The 73% attack rate among those who consumed it can be explained by unknown host characteristics that transmit resistance to the poison, an uneven distribution of the toxin in the meal, or a dose-response relationship. This exact attack rate for the cluster in Riyadh will depend on the detailed community public health outbreak investigation and the total number of cases detected and admitted to other hospitals in the Riyadh region.

Treatment in case of suspected cases of botulism should be initiated immediately to prevent complications. Primary treatment includes supportive in addition to ventilator care and antitoxin. Ideally antitoxin should be given as soon as possible within 24 h [25] without waiting for laboratory confirmation if case is meeting clinical and epidemiological criteria for botulism. Timely antitoxin bounds free toxin in blood and prevent development of symptoms [25], but in case it is delayed like in our study where patients present late to the hospital, wherein symptoms have already set in antitoxin can slow progress of disease and help in recovery by decreasing severity of illness. It has also known to avoid death [26].

## Lessons Learned

The investigation of the botulism outbreak revealed several crucial lessons. Early detection and prompt diagnosis were essential for effective management, as recognizing characteristic symptoms like cranial nerve palsies and descending paralysis significantly improved patient outcomes. The case definition for identifying true cases of botulism is believed to be highly specific, accurately identifying individuals with the disease based on characteristic symptoms and laboratory confirmation. However, its sensitivity may be limited, potentially missing some true cases due to the variability of symptoms and delays in seeking medical care or diagnosis. Rapid administration of botulism antitoxin within the first 48 h of symptom onset played a key role in mitigating disease severity and preventing fatalities. Comprehensive assessment of symptoms, including gastrointestinal issues and neurological impairments, ensured that significant but less obvious symptoms like respiratory distress were not overlooked. Clinicians were advised to maintain a high index of suspicion for botulism, especially in patients with relevant clinical presentations and a history of consuming potentially contaminated food. Effective communication between healthcare providers, public health authorities, and the public was critical for disseminating information about the outbreak source and preventive measures. The importance of food safety through proper handling, preparation, and storage was underscored, highlighting the need for strict adherence to regulations and regular inspections of food establishments. The outbreak emphasized the necessity for healthcare facilities to have protocols for rapid response, including the availability of antitoxin and supportive care capabilities. Continuous education and training for healthcare providers on botulism identification and management were recommended to enhance preparedness and response. Applying these lessons can lead to more effective management of future outbreaks, reducing both the incidence and severity of botulism cases.

## Study Limitation

Our study has certain limitations, including potential recall bias and inaccuracies in reported food consumption, as it relied on retrospective analysis. The small sample size further limits the generalizability of our findings. Additionally, some patients presented late to the hospital, which may have affected the outcomes and the comprehensiveness of the data collected. Blood samples were not obtained before administering the antitoxin, which complicates the assessment of toxin levels and the efficacy of the antitoxin treatment. Moreover, the results of the community outbreak investigation were not included in the analysis of the patients’ hospital admissions, which limits the context and understanding of the outbreak’s broader impact. These limitations highlight areas that could enhance foodborne botulism prevention, surveillance, and early detection. Improving public health education on recognizing early symptoms, ensuring timely presentation to healthcare facilities, and enhancing laboratory capabilities for toxin detection before treatment are essential steps. Additionally, a more robust surveillance system and comprehensive outbreak investigations could provide better data and inform more effective prevention strategies.

## Conclusion

Our analysis revealed a range of symptoms upon hospital admission, including gastrointestinal complaints, cranial nerve palsies, extremity weakness, and respiratory distress. Patients exhibiting cranial nerve signs or symptoms should be assessed by clinicians for respiratory involvement and descending paralysis (i.e., proximal to distal), which may indicate botulism. Most patients arrived at the hospital within 48 h of symptom onset, and those requiring intubation typically received it within the first two days of hospitalization. The absence of fatalities in this patient cohort can be attributed to the prompt detection and treatment of botulism, which was linked to the consumption of contaminated mayonnaise from a well-known local burger establishment.

## Data Availability

No datasets were generated or analysed during the current study.
