# Correction: The First Reported Foodborne Botulism Outbreak in Riyadh, Saudi Arabia: Lessons Learned

**DOI:** 10.1007/s44197-024-00264-y

**Published:** 2024-07-02

**Authors:** Nadeem Gul Dar, Sarah H. Alfaraj, Khulood Naser Alboqmy, Nazia Khanum, Faleh Alshakrah, Hassan Abdallah, Mohammad Hosni Badawi, Ohoud Mohammed Alharbi, Khadijh Ahmed Alshiekh, Abdullah M. Alsallum, Ahmed Hassan Shrahili, Zeidan A. Zeidan, Zaki Abdallah, Ahmed Ali Majrashi, Ziad A. Memish

**Affiliations:** 1 Prevention and Control of Infection Administration, King Saud Medical city, Riyadh, Saudi Arabia; 2 King Salman Hospital, Riyadh, Saudi Arabia; 3 Imam Abdulrahman Alfaisal Hospital, Riyadh, Saudi Arabia; 4 Al Iman General Hospital, Riyadh, Saudi Arabia; 5 Research and Innovation Center, King Saud Medical City, Riyadh, Saudi Arabia; 6 College of Medicine, Al Faisal University, Riyadh, Saudi Arabia; 7 Hubert Department of Global Health, Rollins School of Public Health, Emory, University, Atlanta, USA

**Journal of Epidemiology and Global Health**

10.1007/s44197-024-00255-z

When this article was published, a correction in a heading of Table 2 was left uncorrected by mistake.

Instead of “Dysphagia bring this down with number and %” the column heading should be reduced to. Empty except clinical manifestation Number %

The Original article has been corrected.

The Publisher apologises for this mistake.

